# Optimization of extraction process and solvent polarities to enhance the recovery of phytochemical compounds, nutritional content, and biofunctional properties of *Mentha longifolia* L. extracts

**DOI:** 10.1186/s40643-025-00859-8

**Published:** 2025-03-24

**Authors:** Meryem Tourabi, Khaoula Faiz, Rachid Ezzouggari, Bouchra Louasté, Mohammed Merzouki, Musaab Dauelbait, Mohammed Bourhia, Khalid S. Almaary, Farhan Siddique, Badiaa Lyoussi, Elhoussine Derwich

**Affiliations:** 1 Laboratory of Biotechnology, Conservation and Valorization of Bioresources, Faculty of Sciences, Sidi Mohamed Ben Abdellah University, Fez, Morocco; 2 Laboratory of Biotechnology, Environment, Agri-Food, and Health, Faculty of Sciences Dhar El Mahraz, Sidi Mohamed Ben Abdellah University, Fez, Morocco; 3 Phytopathology Unit, Department of Plant Protection, Ecole Nationale d’Agriculture de Meknès, Km10, Rte Haj Kaddour, BP S/40, 50001 Meknès, Morocco; 4 Unity of GC/MS and GC-FID, City of Innovation, Sidi Mohamed Ben Abdellah University, Fez, Morocco; 5 University of Bahr el Ghazal, Freedowm Stree, Wau, 91113 South Sudan; 6 Laboratory of Biotechnology and Natural Resources Valorization, Faculty of Sciences, Ibn Zohr University, 80060 Agadir, Morocco; 7 Department of Botany and Microbiology, College of Science, King Saud University, P. O. BOX 2455, 11451 Riyadh, Saudi Arabia; 8 School of Pharmaceutical Science and Technology, Tianjin University, Tianjin, People’s Republic of China

**Keywords:** *Mentha longifolia*, HPLC–DAD, Polyphenols, Extraction process, Solvent polarities, Antioxidant capacity, Antimicrobial potential, Bioactive preservative

## Abstract

The current study attempted to evaluate the influence of three extraction processes such as Soxhlet, ultrasound-assisted extraction (UAE,) and cold maceration on one hand and solvent polarities (ethanol 70%, ethyl acetate, and water) on the other hand, vs. phytoconstituents and biofunctional properties of *Mentha longifolia* L. Noteworthy, all extracts were examined in terms of their chemical components, phenolic and flavonoid content, antioxidant and antimicrobial potentials. Notably, high-performance liquid chromatography coupled with array detector analysis (HPLC–DAD) showed the existence of many phenolic compounds. Hydro-ethanol extract (ETOH 70% (v/v)) prepared with maceration and Soxhlet process showed the ultimate rate of phenolic compounds coupled with the most powerful antioxidant and antimicrobial capacity. Notably, ETOH extract obtained with UAE showed important nutritional properties, particularly soluble carbohydrate (50.1 ± 0.70 mg/g dry weight), and soluble protein (26.5 ± 0.5 mg/g dry weight), while aqueous extract obtained by use of UAE extraction maximized pigment content. The outcome of this work showed that Soxhlet and maceration extraction processes with many polar solvents are more appropriate for *M. longifolia* extraction. *M. longifolia* possesses promising phytochemicals, which can be used in different sectors like food, pharmaceutical, and cosmetic formulations.

**Graphical Abstract:**

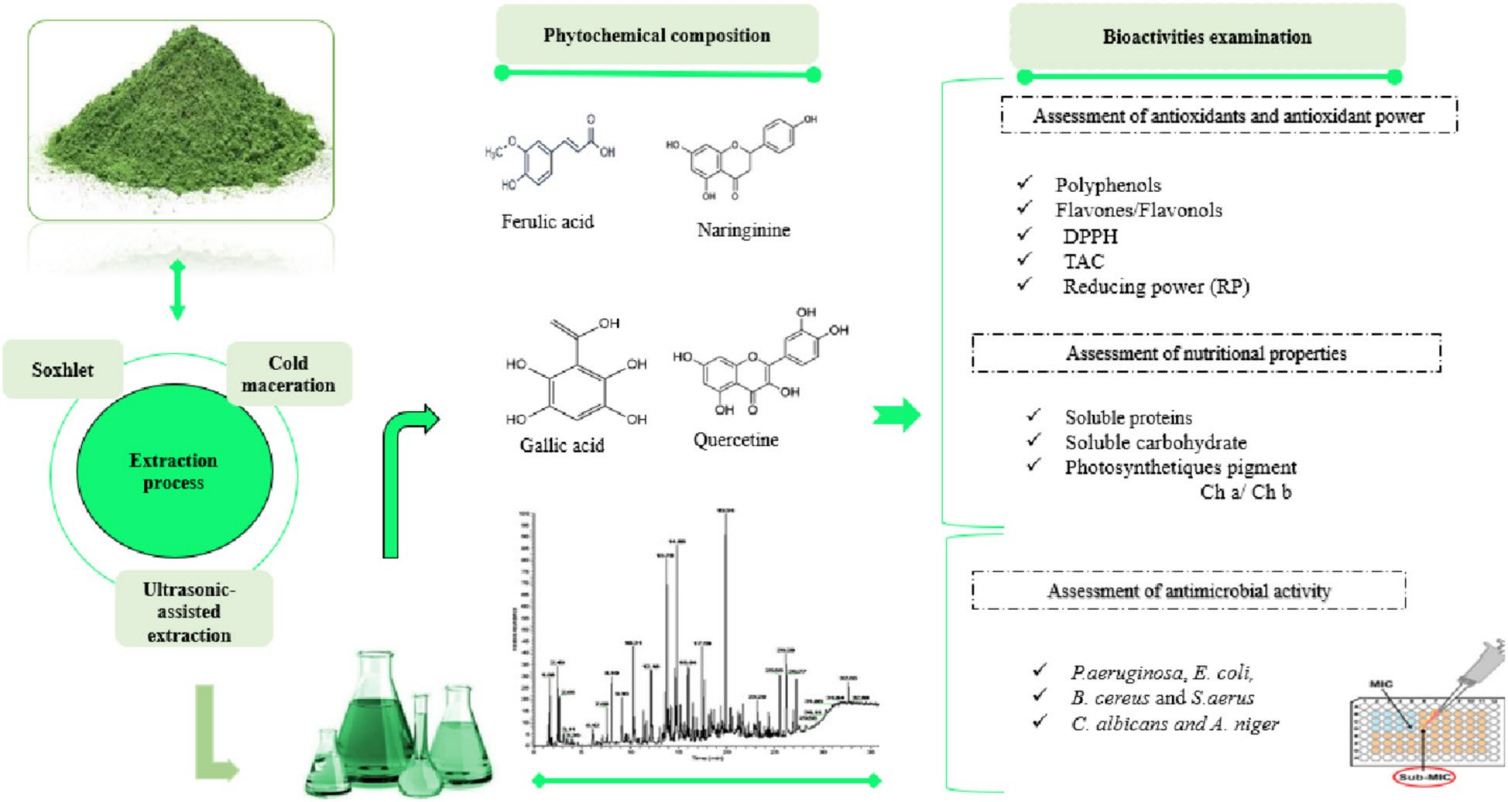

## Introduction

Food preservation is a process aimed at maintaining its quality and safety by countering the harmful effects induced by its natural characteristics or by the presence of microbial contaminants. Various strategies have been developed to reduce these problems, of which the use of preservatives is a commonly employed method. Due to concerns over safety and toxicity associated with synthetic preservatives, as well as increased consumer awareness, the demand for natural products and food preservatives is rising. For this reason, current food preparation techniques are increasingly replacing conventional synthetic additives with natural plant-based ones that are safe from a toxicological perspective.

*Mentha longifolia* L. is a medicinal plant used in traditional medicine thanks to its richness in potential phytochemicals with pharmaceutical properties. Notably, previous research reported the use of plant extracts with bioactive compounds as antibacterial and antioxidant agents. Noteworthy, plant extracts were found to be used as food preservatives (Bondi et al. 2017; Bouarab Chibane et al. 2019).

*Mentha longifolia*, known as 'naana touil' in Morocco, demonstrates these properties due to its abundance of bioactive metabolites, such as tannins, flavonoids, phenolic acids, and volatile compounds. Additionally, it serves as a valuable source of essential nutrients, including proteins, minerals, and vitamins B2 and C (Khan et al. 2011; Hutsol et al. 2023), as well as powerful antioxidants like caffeic acid, quercetin, kaempferol, and cinnamic acids (Tourabi et al. 2023a). These bioactive components provide the therapeutic benefits of *M. longifolia*, which include antimicrobial, antioxidant, anti-inflammatory, antidiabetic, and hepatoprotective activities (Farzaei et al. 2017), making it a cornerstone in traditional medicine for treating gastrointestinal, menstrual, and respiratory conditions. Furthermore, its diverse phytochemical profile highlights its role as a valuable source of therapeutic agents, especially for tackling oxidative stress and bacterial infections.

The choice of extraction techniques and solvent type plays a pivotal role in preserving and optimizing the yield of bioactive compounds from *M. longifolia*, thereby influencing their antioxidant and antibacterial activities. Techniques such as cold maceration, Soxhlet, and sonication have been employed due to their efficacy, simplicity, and adaptability across pharmaceutical, food, and cosmetic industries (Nuzul et al. 2022). These methods allow the efficient recovery of key phytochemicals, such as hydroxycinnamic acids (e.g., rosmarinic, caffeic, and ferulic acids), hydroxybenzoic acids (e.g., gallic and vanillic acids), and flavonoids (e.g., quercetin, kaempferol, luteolin). The documented therapeutic and pharmacological contributions of their group are consistent with the observed bioactivities of *M. longifolia*. Importantly, optimizing extraction conditions, including solvent polarity and temperature, ensures minimal degradation and maximum yield of these bioactive constituents (Gil-Martín et al. 2022). This study highlights the importance of *M. longifolia* as a model plant for achieving high-quality extracts with superior antioxidant and antibacterial properties, thereby reinforcing its potential applications in preserving food products and advancing nutraceutical and pharmaceutical innovations.

Despite the growing interest in environmentally friendly extraction techniques and their impact on bioactive compounds, there is a deficiency of comprehensive data regarding the influence of different extraction methods and green solvents on the phytochemical composition, antioxidant potential, and antimicrobial properties of Moroccan *Mentha longifolia*. Therefore, this study aimed to assess the efficacy of various extraction techniques (Soxhlet extraction, ultrasound-assisted extraction, and cold maceration) and solvents in enhancing the phytochemical profile, antioxidant capacity (as measured by DPPH, reducing power, and total antioxidant capacity assays), and antimicrobial activity of this plant. The ultimate goal is to identify optimal extraction methods and solvents to produce high value extracts with potential applications in the food and pharmaceutical industries. The outcome of this work will provide biochemical evidence for the standardization, safety, and quality of *M. longifolia*, which can then be used in the pharmaceutical and cosmetics industry, and also used in food preservation thanks to its high content of antioxidant phytoconstituents and antimicrobial capabilities.

## Material and methods

### Chemicals and reagents

Ethanol (90%), ethyl acetate, 2, 2-diphenyl-1-picrylhydrazyl (DPPH), sodium phosphate buffer, potassium ferricyanide, trichloroacetic acid (TCA), ferric chloride (FeCl_3_), ammonium molybdate, sulfuric acid(H_2_SO_4_), sodium phosphate, Folin-Ciocalteu reagent, aluminum trichloride (AlCl_3_), sodium carbonate (Na_2_CO_3_), copper sulfate (CuS04), sodium potassium tartrate, bovine serum albumin (BSA), ascorbic acid, phenol reagent, glucose, sodium hydroxide (NaOH), dimethyl sulfoxide (DMSO 2%), MH (Mueller–Hinton), Sabouraud Dextrose (SDA) were purchased from Sigma-Aldrich Chemical Co. (United States of America,), resazurin, rutin, quercetin, catechol, kaempferol, syringic acid, gallic acid, pyrogallic acid, ferulic acid, rosmarinic acid, vanillic acid, coumaric acid caffeic acid, protocatechuic acid was obtained from Abcam, United Kingdom. All used chemicals were of analytical grade.

### Collection of plant material

*Mentha longifolia* L. plant used in our study was collected between June and October 2021 (flowering period) from the town of Ifran (latitude 33°31ʹ35ʺN; longitude 5°06ʹ 36ʺ; altitude 1648 m). Professor Amina BARI, a botanist at Sidi Mohamed Ben Abdellah University, identified a voucher specimen in the faculty herbarium under the number (001MLAV202162).

### HPLC–DAD analysis

The phytochemical composition of *M. longifolia* extract has been determined using an array detector for reverse-phase high-performance liquid chromatography following the protocol described by Tourabi et al., (Tourabi et al. 2023a). Every phenolic substance, including rutin, quercetin, catechol, kaempferol, syringic acid, gallic acid, pyrogallic acid, ferulic acid, rosmarinic acid, vanillic acid, coumaric acid caffeic acid, protocatechuic acid has been determined by considering the duration of its retention with that of standards compounds.

### Extraction procedure

The aerial parts of *Mentha longifolia* were mechanically ground using a China Tencan 15L inner-lined blender operating at 6000 rpm. The grinding process was carried out for a total duration of 4 min, with intermittent pauses of 15 s after every 30 s of operation. Extractions were performed with three analytical solvents (ethanol 70%, ethyl acetate, and distilled water), followed by three procedures.

### Soxhlet extraction

The extraction of compounds from the powder of *M. longifolia* is performed by using the Soxhlet procedure cited by Alara et al. (Alara et al. 2018). Ten grams of the aerial part of the matrix was weighed and placed in a filter paper cartouche, which is placed, inside the Soxhlet apparatus cavity. 70% Ethanol, ethyl acetate, and distilled water can be placed in a 250 mL conical flask according to the solvent supply. A heating flask was used to heat the mixture to reflux, and the extraction time was between 1 and 4 h. The extracts were filtered using a cone-shaped filter paper (Whatman No. 01), and the achieved filtrate was centrifuged at 1680 rpm for 10 min. After that, the supernatant was evaporated at 40 °C and 250 to 280 rpm using a rotating vacuum evaporator (BUCHI Rotavapor R­200) under decreased pressure. Afterward, they were stored at 20 °C in a refrigerator until used to assess their biological activity.

### Ultrasonic-assisted extraction (UAE)

The extraction of compounds from the powder of *M. longifolia* is performed by using the ultrasonic-assisted extraction (UAE) process described by Dhanani et al. Briefly, and tree solvents (70% ethanol, ethyl acetate, and distilled water). The ideal extraction condition is a sample/solvent ratio, of 1:20, 20 min of extraction time, and 25 °C of temperature for extraction in an ultrasonic bath at a frequency of 40 kHz (Dhanani et al. 2017). The extract obtained after sonication was filtered into a cone of filter paper (Whatman N°01), and the achieved filtrate was centrifuged at 1680 rpm for 10 min, and the supernatant was then evaporated under reduced pressure operating a rotating vacuum evaporator (BUCHI Rotavapor R­200) at 40 °C and 250 to 280 rpm. The dried residue was subsequently stored at − 20 °C until further use. The extracts obtained through Ultrasonic-assisted extraction were evaporated to obtain a dry mass, which was then used to evaluate their biological activity.

### Cold maceration

Maceration is a conventional method recently used in medicinal herb research and manufacturing enterprises. Briefly, *M. longifolia* powders were extracted by the maceration process as described by Azwanida et al., (Azwanida 2015). One gram of the dried and ground aerial part of the vegetable matrix was soaked in 20 mL of each solvent (70% ethanol, distilled water, and ethyl acetate), for 72 consecutive hours at ambient temperature in dark conditions with continuous shaking. The final extracts obtained after maceration were filtered into a cone of filter paper (Whatman N°01), evaporated by rotary vapor dry mass at 250 to 280 rpm, then used to evaluate their biological activity, and stored at ­20 °C.

In a rotating evaporator operating under vacuum (BUCHI Rotavapor R­200), the solvents and water were evaporated at 250 to 280 rpm and 40 °C and the resulting crude extracts were weighed to determine the yield. Y(extract) (%) is the percentage of dry matter extracted, defined by the following equation (1).

1 $${\text{Y}}{\mkern 1mu} {\mkern 1mu} \left( {{\text{extract}}} \right){\mkern 1mu} {\mkern 1mu} \left( \% \right) = \frac{{{\text{ Weight of dry extract }}\left( {\text{g}} \right)}}{{{\text{Weight of dry plant }}\left( {\text{g}} \right)}} \times 100$$

### Free radical scavenging (DPPH test)

The radical-scavenging capacity of each extract against the 2, 2-diphenyl-1-picrylhydrazyl (DPPH) free radical was measured using the free radical scavenging assay (DPPH) the antioxidant activity was determined following the procedure described by Brand-Williams et al., (Brand-Williams et al. 1995). Eight hundred and twenty-fifth microliter of the ethanolic reagent of DPPH (prepped in 70% ethanol, at 517 nm, with an absorbance of 0.700 and a molarity of 60 μM) have been added to twenty-five microliters of each extract. Absorbance measurements are measured at a wavelength of 517 nm after a 60-min incubation in dark conditions. The IC_50_ was determined from the graph by the percentage of inhibition using the following equation (2).

2 $${\text{Inhibition }}\left( \% \right) \, = \, \left[ {\left( {{\text{Abs}}_{{{\text{control}}}} - {\text{ Abs}}_{{{\text{sample}}}} |{\text{Abs}}_{{{\text{control}}}} } \right) \, \times { 1}00} \right]$$

### Reducing antioxidant power (RP test)

By following the method outlined by Padmanabhan et al., the reducing antioxidant power (RP) test was carried out (Padmanabhan and Jangle 2012). This procedure is based on converting Fe2 + ions into Fe3 + by the antioxidant phenolic biomolecules. Each extract was mixed with 50 µl of 0.2 M sodium phosphate buffer (pH 6.6) and 250 μL of 1% potassium ferricyanide. For twenty minutes, the mixture was placed in a water bath at 50 °C, after 250 μL of 10% trichloroacetic acid (TCA), 250 μL of distilled water, and 60 μL of 0.1% ferric chloride (FeCl_3_) were included. Measurements of absorbance were conducted at a wavelength of 700 nm. The values of the extract's reductive power are described as milligrams of ascorbic acid equivalent (AAE) per gram of dry weight (mg AAE/g DW). Performed all Tests in triplicate.

### Total antioxidant capacity

The ammonium molybdate approach previously mentioned by Prieto et al., was used to determine the total antioxidant activity (TAC) (Prieto et al. 1999). Briefly, 1 mL of reagent solution (4 mM ammonium molybdate, 0.6 M sulfuric acid, and 28 mM sodium phosphate), was mixed with twenty-five microliters of each extract. The absorbance was measured after under 95 °C in a thermal block for 90 min, then absorbance was measured at 695 nm relative to a blank. A calibration curve is formulated using a standard solution of ascorbic acid, and TAC values are represented as milligrams of ascorbic acid equivalent (AAE) per gram of dry weight (mg w/g DW).

### Phytochemical content

#### Total phenolic content (TPC)

The Folin–Ciocalteau protocol described by Rouphael et al. was used to determine the total phenolic component in the plant samples (Rouphael et al. 2016) with slight modifications. Gallic acid (0.024 –1.061 mg/mL) was served as a reference to create the calibration grape (R^2^ = 0,999), and the phenolic components were expressed as milligrams of gallic acid equivalent (GAE) per gram of dry weight (mg GAE/g DW), performed all tests in triplicate.

### Total flavonoid Content (TFC)

The aluminum chloride assay cited by San et al. was assessed to estimate the flavone, and flavonol content (San et al. 2013). Quercetin (0.138 – 1.114 mg/mL) has been used as a standard to establish the curve (R^2^ = 0.994). The results obtained are expressed as milligrams of quercetin equivalent (QE) per gram of dry weight (mg QE/g DW), performed all tests in triplicate.

### Nutrient determination

#### Soluble proteins content

The approach provided by Lowry et al. was used to estimate the soluble protein amount (Lowry 1951), with some slight changes. In brief, 0.1 mL of plant extract was combined with 1 mL of Lowry coloring reagent, and 100 μL of Folin reagent (1/3) was added. The results were placed in the dark for 30 min using a spectrophotometer adjusted to measure absorbances at 750 nm. Bovine serum albumin (0.002 – 0.116 mg/mL) was employed as a standard for preparing a calibration curve (R^2^ = 0.9906) and the results were given in milligrams (mg BSAE/g DW), or BSA equivalents per gram of dry matter.

### Chlorophyll pigment content

The spectrophotometry protocol stated by Sumanta et al. was used to estimate the amount of chlorophyll-a and chlorophyll-b in our extracts (Sumanta et al. 2014). For this reason, a 2 mL extract was centrifuged at 10,000 rpm until 15 min. Filtrate and a spectrophotometer were collected to determine the absorbances at 649 and 664 nm. The quantification of pigment chlorophyll – a (Ch – a) and chlorophyll – b (Ch – b), was achieved using the formula (3) and (4).

3 $${\text{Ch }}{-}{\text{ a }} = { 13}.{\text{36Abs}}_{{{\text{664nm}}}} - { 5}.{\text{19Abs}}_{{{\text{649nm}}}}$$

4 $${\text{Ch }}{-}{\text{ b }} = { 27}.{\text{43Abs}}_{{{\text{664nm}}}} - { 8}.{\text{12Abs}}_{{{\text{649nm}}}}$$

For each photosynthetic pigment, the data were represented as milligrams (mg Ch – a or Ch –b/g DW) of the dried plant.

### Total soluble carbohydrate content

The phenol–sulfuric acid procedure outlined by Masuko et al. was used to measure the total carbohydrate rate (Masuko et al. 2005). To proceed, 150 μL of sulfuric acid (96–98% (v/v)) was added to 50 μL of every sample. Next, add 30 μL of the phenolic reagent (5%) and the last mixture was warmed to 90 °C for 5 min. After cooling for five minutes to ambient temperature, the absorbances were taken using a spectrophotometer at the wavelength of 490 nm. The curve for calibration was created using glucose as the reference value (R^2^ = 0.992). The total carbohydrate content was represented as milligrams of glucose equivalents (GlcE) per g of the dry plant (mg GLcE/g DW).

### Antimicrobial sensitivity tests

We assessed the antimicrobial potency of our extracts against four different bacterial strains: two Gram-negative bacteria including* (Escherichia coli*, K12, and *Pseudomonas aeruginosa* CIP A22*)* as well as against two Gram-positive strains, notably (*Staphylococcus aureus* ATCC 6633, and *Bacillus subtilis* DSM 6333); and two fungal strains, such as (*Candida albicans*, ATCC 10231, and *Aspergillus niger*, MTCC 282). (University Hospital Complex, Fez, Morocco), all tested strains have been obtained clinically. Multi-drug resistance was identified in these bacterial and fungal species.

### Minimum inhibitory concentration (MIC)

The approach of microdilution cited by Bachiri et al. was applied to determine the MIC effects of *M. longifolia* aerial part extracts against various multidrug strains (Bachiri et al. 2016) (Oreopoulou et al. 2019). One hundred microliters of each extract (50 mg/ml), in microplate wells, sterile MH broth for bacteria and SDA for fungi, and 50 μL of microbial inoculate were combined for every concentration in the dilution succession, achieving a final microbial concentration of 10^8^ CFU/mL. Next to a 48- to 7-night incubation time at 37 °C for fungi and a 24-h interval for bacteria (Ozkan et al.^2021^), the colorimetric mean was utilized to determine the minimum inhibitory concentration (MIC) using resazurin 0.015% (Yusoff et al. 2022).

### Minimum bactericidal concentration (MBC)

The broth micro-dilution method outlined by Bachiri et al. was applied to determine the minimal concentration of bactericide (MBC) (Bachiri et al. 2016). Briefly, 3 μL of the suspension from each well was transferred into MH agar plates where no noticeable growth was seen. After that, the dishes were incubated at 37 °C for a total of 24 h.

### Statistical analysis

The statistical study, which included a One-way ANOVA test, was conducted using GraphPad Prism 8. Principal component analysis (PCA) was performed using RStudio software; a variation of p < 0.05 was considered significant. Two-way interaction linear nonparametric models were constructed to assess the primary impacts of the extraction method, solvent, and their interactions on all variables (assay).

## Results and discussion

### Effect of solvent types and extraction techniques on extraction yield

**Fig. 1:**
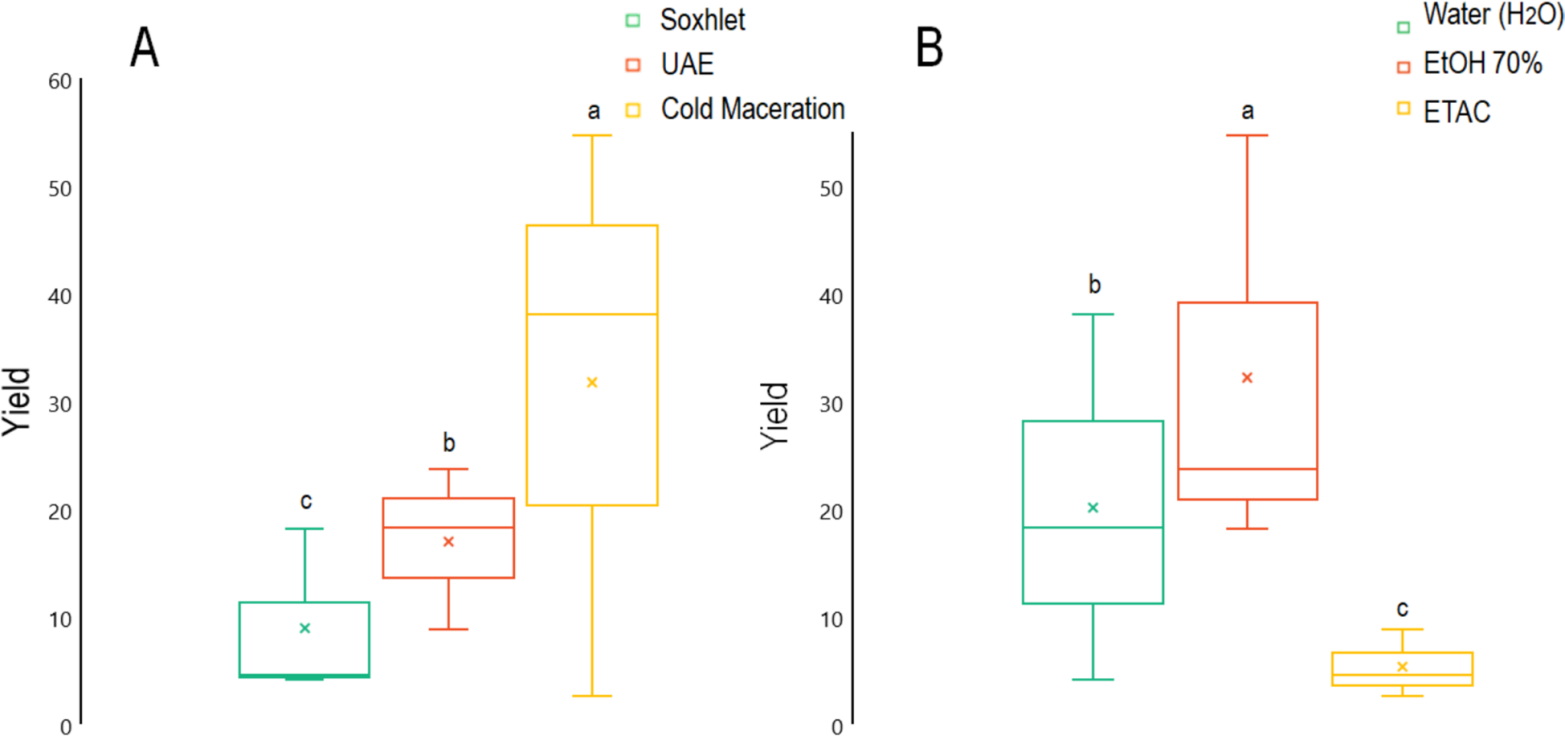
Impact of solvent varieties and processes of extraction on *M. longifolia* yields of extraction. **A**: Extraction process; **B**: Solvent types

To achieve substantial yields, the ideal extraction process and solvent combination must be selected based on the characteristics of the matrix. Water, ethanol, and ethyl acetate were utilized as extraction solvents. Processes for extraction including Soxhlet, Ultrasonic assisted extraction (UAE), and cold maceration were also examined on the extraction yield. The yield outcomes of various *M. longifolia* extracts are displayed in Table 2 and Fig. 1. According to the data, cold maceration extraction produced the highest extraction yield in combination with 70% EtOH, followed by UAE, however, Soxhlet achieved the lowest yield when 70% ethanol was added. This result is in arrangement with those stated by Krakowska-Sieprawska (Krakowska-Sieprawska et al. 2020).

### Effect of solvent types and extraction techniques on phenolic composition of extracts

To further investigate the impact of solvent polarities and extraction process to recover the phenolic composition of the extracts, we conducted a tentative quantification and identification of components using HPLC–DAD, resulting in the identification of thirteen compounds in this analysis (Table 1). The findings revealed that all extracts were characterized by different compounds with various concentrations. Regarding Soxhlet extraction, the data indicates the presence of all phytoconstituants distributed among the three solvents (water, ETOH 70%, and ETAC). Quercetin was detected at 7.30 and 11.97 mg/g DW in the ethyl acetate extract obtained by sonication (UAE) and Soxhlet extraction. Rutin was found at a high level only in ETOH70% obtained by cold maceration technique 7.34 mg/g DW, as well as in ETAC extract obtained by the Soxhlet method with a concentration of 4.95 mg/g DW. Catechol was detected only in extract achieved by the Soxhlet process, notably the high concentration noticed in aqueous extract 3.03 mg/g DW pursued by ETOH 70% with a concentration of 2.61 mg/g DW. However, the last one was undetected in other extracts. Kaempferol is found in a high amount in the ETAC extract 3.25 mg/g DW produced by cold maceration method. In addition, vanillic acid was detected at a high concentration ranging between 3.41 and 4.11 mg/g DW in EtOH 70% extract obtained by Soxhlet and sonication process respectively. Coumaric acid is quantified at a high level only in the ultrasonic extracts, notably in ETOH 70% 8.84 mg/g DW, while undetected in other extracts. Gallic acid is the major phenolic acid found in the ETAC extract 11.03 mg/g DW achieved by cold maceration process. Rosmarinic acid is detected in the high concentration, especially in the ETAC extract 3.03 mg/g DW obtained by cold maceration technique. The ETAC extract achieved by sonication shows a high amount of ferulic acid 8.21, whereas the aqueous extract prepared with cold maceration indicates a middle amount of 4.20 mg/g dw.

**Table 1:** Estimation and identification of the individual phenolic components present in the various *M. longifolia* extracts

	Extraction Process /Solvent								
	Soxhlet			Ultrasonic assisted extraction (UAE)			Cold maceration		
	Water	EtOH 70%	ETAC	Water	EtOH 70%	ETAC	Water	EtOH 70%	ETAC
Hydroxycinnamic acids (mg/g dw)									
Rosmarinic acid	ND	ND	2.09	0.13	ND	2.33	ND	0.24	3.03
Coumaric acid	ND	ND	ND	0.03	8.84	ND	ND	ND	ND
Caffeic acid	3.50	1.54	0.78	0.06	1.33	ND	2.85	ND	ND
Ferulic acid	0.58	ND	0.88	0.49	ND	8.21	4.20	ND	2.27
Hydroxybenzoic acids (mg/g dw)									
Gallic acid	0.29	1.63	ND	0.91	ND	0.10	ND	ND	11.03
Pyrogallol	0.09	0.17	1.63	6.78	1.02	0.11	6.84	ND	0.67
Vanillic acid	2.00	3.41	3.63	0.04	4.11	ND	ND	2.71	2.76
Protocatechuic acid	1.04	0.83	ND	0.30	ND	1.93	0.31	0.52	ND
Syringic acid	ND	0.03	0.13	0.08	0.22	1.30	ND	ND	ND
Flavonoids (mg/g dw)									
Rutin	1.13	1.30	4.95	0.29	0.40	ND	ND	7.34	ND
Quercetin	3.91	0.77	11.97	ND	1.09	7.30	0.06	3.18	ND
Catechol	3.03	2.61	1.42	ND	ND	ND	ND	ND	ND
Kaempferol	2.88	1.10	0.71	0.11	ND	0.52	0.72	0.52	3.25

*ETAC* Ethyl acetate, *ND* not detected

In addition, the choice of solvent polarities and extraction process, bioavailability of bioactive composites, type of matrix as well as time and temperature of extraction were the most significant parameters affecting and illustrating the notable variations in the recovery of polyphenols from different analyzed samples (Pequeño Alonso 2022). The variation in the phenolic profile of extracts indicates the interaction effect amongst plants, solvent type, and extraction process. Silva and coworkers did not identify vanillic, syringic acid, and kaempferol in most test extracts which was the opposite of the case in our study, depending on the extraction process and solvent type used (Silva et al. 2021).

Kivilompolo a colleague, also studied the identification of polyphenol compounds from spearmint and other plant extracts. Similarly, to the results we found in our research, the authors found rosmarinic acid in spearmint 5620 μg/g DW and also found vanillic acid, syringic, coumaric, and ferulic acids across all herbs extracts. In agreement, Kivilompolo et al. reported that spearmint contained chlorogenic acid 310 μg/g DW, while the last one was never detected in extracts in our investigation (Kivilompolo and Hyötyläinen 2007).

In summary, except for chlorogenic acid, resveratrol, and ellagic acid, our outcomes are consistent with those of Silva et al., since certain of the chemicals identified in those studies were also found in various of our extracts (Silva et al. 2021).

In this sense, various solvents have successfully recovered diverse plants' polyphenols. Mixtures of alcohols (MeOH, EtOH) with water are frequent solvents in the extraction of bioactive molecules for phytochemistry studies (Hikmawanti et al. 2021). Since it is less harmful, it can be applied instead of the later method for using the recovered polyphenols in food or cosmetic applications. On the other hand, distinguished polyphenol class fractions can be recovered by consecutive extraction using solvents with higher polarity (Oreopoulou et al. 2019).

Aqueous ethanol has been reported to be more effective than pure alcohols as a solvent for extracting hydroxycinnamic acids, such as rosmarinic and caffeic acid, a result that aligns with our findings (Oreopoulou et al. 2019).

It is important to note that solvent polarity influences not only the extraction efficiency but also the spectrum of bioactive compounds extracted. Thus, Soxhlet extraction using ethanol has been reported to extract a broad range of phytochemicals, including alkaloids, flavonoids, and saponins. This may be due to a synergy between the solvent and the prolonged exposure to heat (Ozkan et al. 2021). In addition, UAE enhances the extraction of polyphenolic compounds due to its ability to break down cell walls and improve solvent penetration (Yusoff et al. 2022). Literature reports that cold maceration is effective for extracting thermolabile compounds, such as polyphenols, without the risk of degradation from heat. However, its efficiency can be limited by the solvent's ability to diffuse into the plant cells and by the extraction time required (Farooq et al. 2022).

### Effect of solvent types and extraction techniques on Total phenolic and flavonoids content

Polyphenols are secondary plant metabolites present in all parts of plants (stems, flowers, leaves, roots, fruits, seeds, and wood), and they are often found in plant foods (Tanase et al. 2019). Over 8,000 phenolics are known and identified in plants. The most important categories are phenolic acids, phenolic amides, lignans, stilbenes, and flavonoids (Cheynier 2005). Moreover, flavonoid compounds are the most heterogeneous class of phenolic compounds, some being soluble in apolar solvents. These phytocomponents contribute to numerous biological potentials and are protected from chronic diseases, namely those related to oxidative stress, including diabetes cardiovascular illness, cancer, and chronic inflammation (Reddy and Prabhakaran 2020). Furthermore, the polarity of the solvent and the extraction procedure are critical factors affecting the quality of the extract composition (Thouri et al. 2017). In our experiment, three analytical solvents of increasing polarity were selected for the extraction of phenolic components from the aerial part of *M. longifolia*, notably ethyl acetate (polarity 4.4), ethanol (polarity 5.2), and water (polarity 10.2). As mentioned in Table (2), our results show that the extract obtained with 70% ethanol presents a higher level of flavonoid and phenolic amounts than those obtained with water and ethyl acetate for all tested techniques. In addition, the polyphenol and total flavonoid contents are significantly varied depending on the solvent and method of extraction selected, where the hydroethanolic extracts obtained by Soxhlet extraction have the highest amounts of phenolic compounds followed by the same extract obtained by cold maceration, and ultrasound-assisted extraction (76.7 ± 0.01, 71.4 ± 0.6, and 68.6 ± 0.1 mg GAE/g DW respectively), in the opposite for sonication technique with water and ethyl acetate solvent showed the lowest amount of total phenolic content. These outcomes were better than the ones stated by Bourgou et al., who were interested in hydroethanolic extracts of *Euphorbia helioscopia*, and reported that the TPC contents ranged between 23.70 and 44.45 mg GAE/ g DW (Bourgou et al. 2016). Likewise, Farahani et al. evaluated the influence of extraction technique and solvent types on the rate of phenolic content of *Arctium lappa* L. roots and *Polygonum aviculare* L, and the results indicate that the ethanol extract obtained by the Soxhlet method showed a significant effect on maximizing the extraction of phenolics content (Farahani 2021). Accordingly, our results were in line with those reported recently for the extracts of *Arctium lappa* roots, the higher rate of phenolic content was given for the Soxhlet extraction with hydroethanolic solvent (72.61 mg GAE/g DW), and dichloromethane (79.45 mg GAE/g DW) (Bourgou et al. 2016).

Regarding flavonoid content, cold maceration with 70% ethanol was especially effective in the recovering of flavonoid content with a rate of 21.9 ± 0.1 mg QE/ g DW, followed by Ultrasonic assisted extraction and Soxhlet extraction with the same solvent with a value of 16.9 ± 1.1, 14.8 ± 0.6 mg QE/ g DW respectively. In contrast, the lowest flavonoid content was observed in Ultrasonic-assisted extraction with ethyl acetate (3.2 ± 0.3 mg QE/g DW), followed by Soxhlet extraction using water (6.94 ± 0.2 mg QE/g DW) and ethyl acetate (5.4 ± 0.7 mg QE/g DW), as shown in Table 2. Moreover, they report lower flavonoid compounds than our sample which have higher contents, ranging from 5.66 to 30.70 mg QE/ g DW (Bourgou et al. 2016). In addition, research performed by Alara et al. indicates that Soxhlet extraction with an ethanol concentration of 60% v/v provides the best TFC with a rate of (30.0 ± 0.4 mg QE/g d.w.) (Alara et al. 2018). These studies have indicated that an ethanol concentration of 70% extracts phenolic and flavonoid compounds more effectively than water and ethyl acetate.

**Table 2:** Summary statistics (mean ± standard error) for antioxidant content and assay of extracts by technique and solvent were calculated

Extraction technique	Solvent type	Yield (%)	Polyphenols (mg GAE/g DW)	Flavonoids (mg QE/g DW)	DPPH (mg/mL)	RP (mg/mL)	TAC (mg AAE/g DW)
Soxhlet	H_2_O	4.1 ± 0.02^b^	59.9 ± 0.4^b^	6.94 ± 0.2^b^	0.1 ± 0.0^b^	0.25 ± 0.01^b^	53.53 ± 0.28^b^
	EtOH 70%	18.2 ± 0.0^a^	76.7 ± 0.01^a^	14.8 ± 0.6^a^	0.05 ± 0.00^a^	0.11 ± 0.01^a^	94.1 ± 1.40^a^
	ETAC	4.5 ± 0.04^b^	51.4 ± 0.4^b^	5.4 ± 0.7^b^	0.1 ± 0.0^b^	0.21 ± 0.01^b^	90.3 ± 1.7^a^
Ultrasonic assisted extraction (UAE)	H_2_O	18.2 ± 0.2^b^	31.9 ± 0.8^b^	11.8 ± 0.9^b^	0.1 ± 0.0^b^	0.15 ± 0.00^b^	77.1 ± 0.6^b^
	EtOH 70%	23.5 ± 0.7^a^	68.6 ± 0.1^a^	16.9 ± 1.1^a^	0.1 ± 0.0^a^	0.07 ± 0.00^a^	88.8 ± 1.8^a^
	ETAC	8.8 ± 0.1^c^	15.9 ± 0.4^c^	3.2 ± 0.3^c^	0.74 ± 0.01^c^	1.51 ± 0.01^c^	35.5 ± 1.0^c^
Cold maceration	H_2_O	37.8 ± 1.1^b^	35.2 ± 0.4^b^	11.1 ± 0.6^b^	0.2 ± 0.0^b^	0.34 ± 0.01^b^	55.7 ± 0.6^b^
	EtOH 70%	54.0 ± 1.4^a^	71.4 ± 0.6^a^	21.9 ± 0.1^a^	0.03 ± 0.00^a^	0.08 ± 0.01^a^	93.85 ± 0.01^a^
	ETAC	2.5 ± 0.0^c^	36.2 ± 0.1^b^	7.9 ± 0.1^c^	0.2 ± 0.01^b^	0.40 ± 0.01^b^	44.2 ± 0.3^c^

*SP* Soluble proteins

This can be attributed to the accessibility of phenolic molecules in a solvent, the chemical composition of the plant material, the pH of the extraction solvent, the polarity of the chosen solvent (Tomšik et al. 2016), as well as the temperature of extraction can influence positively or adversely the extraction of thermolabile compounds (Wong et al. 2014; Lee and Petersen 2003). Polyphenols are very hydrophilic due to their phenolic nature, water, polar organic solvents, notably acetone, methanol, ethanol, and acetonitrile, or their mixtures of water, are used to extract the polyphenol compounds including aglycones, glycosides, and oligomers (Tsao 2010). These findings are significant as they aid in the identification of the optimal method and solvent combination based on the specific phenolic compound one aims to extract from the sample.

Additionally, we incorporate interaction terms into the models to show how the proportion of one or more other variables affects a factor's influence. In this case, it would not be fair to extrapolate the trends shown by the primary impacts without accounting for the interactions if they show statistical significance.

**Fig. 2:**
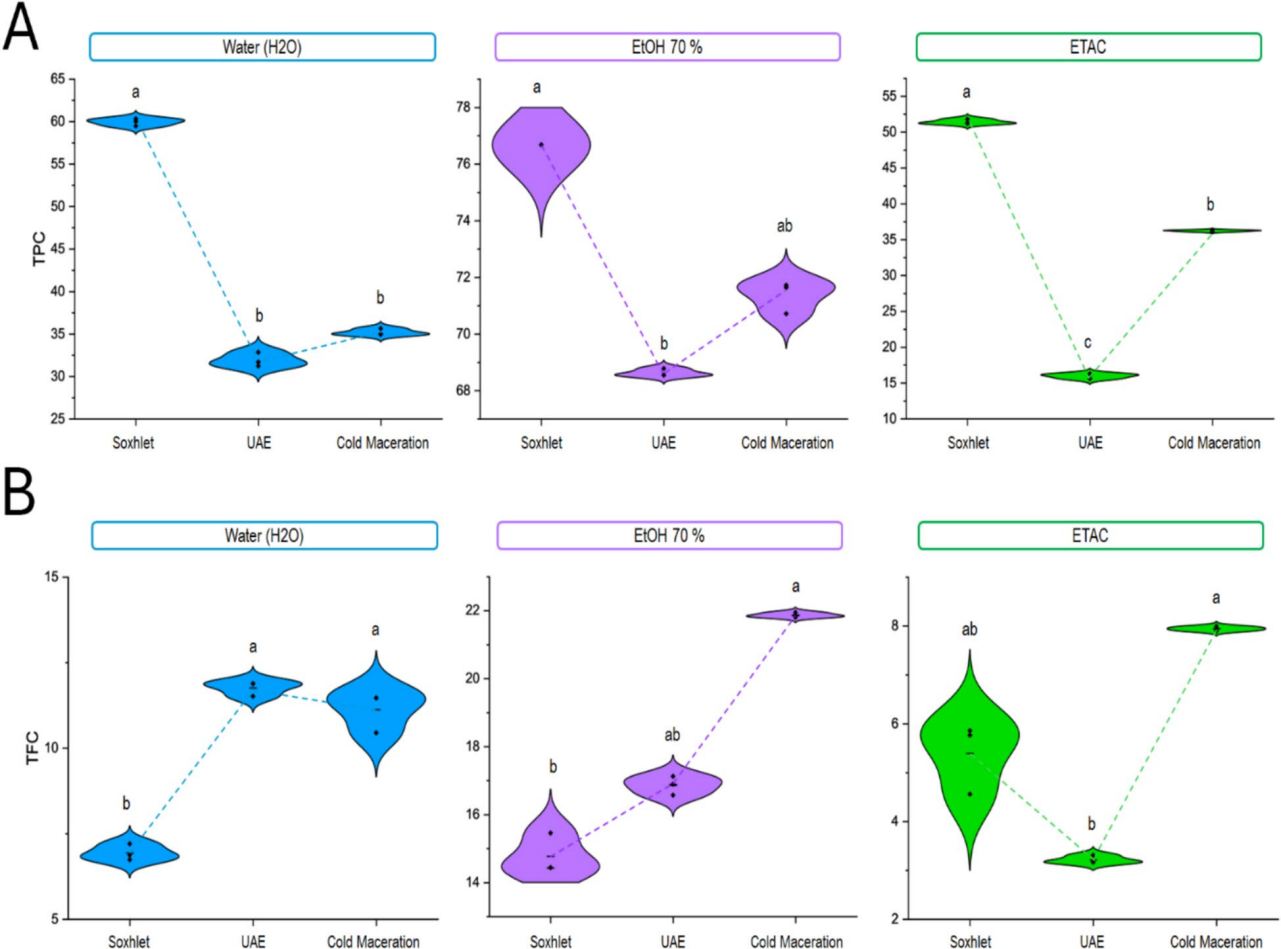
Graphs of the interaction between “solvent × method” on the total content of **A** phenolic and **B** flavonoid (TFC) in *M. longifolia* extracts. *ETOH 70%* Hydro-ethanol, *ETAC* ethyl acetate, *UAE* ultrasonic-assisted extraction

The stated interaction effects “method × solvent” on TPC and TFC amount of different *M. longifolia* extracts is displayed in Fig. 2. Considering this, as well as the outcomes shown in Table 2. However, Soxhlet extraction, maceration, and ultrasonic extraction using 70% (v/v) ethanol, water, and ethyl acetate as solvents are solvent combinations that can be chosen for their capacity to yield extracts with high levels of flavonoids and phenolic compounds as well as strong antioxidant properties.

Figure 2 presents each violin plot in the distribution of TPC and TFC values ​​obtained from three extraction methods, for each solvent combination, and the results indicate that it is evident that the solvent type has a very different on the TPC and TFC content. Soxhlet extraction using 70% ethanol is an efficient method in analytical chemistry, allowing for the extraction of polar compounds like phenolics and flavonoids, resulting in higher bioactive compound yields. Cold maceration extraction using 70% ethanol (v/v) is effective for extracting phenolic and flavonoid compounds, yielding significant amounts of bioactive compounds without excessive degradation of heat-sensitive compounds, despite being slower than Soxhlet extraction.

In summary, the extraction process employs the Soxhlet process combined with 70% ethanol (v/v) as solvent and cold maceration extraction using 70% ethanol (v/v) that may be adopted for their effectiveness in producing extracts with higher phenolic and high flavonoid contents.

### Effect of solvent types and extraction techniques on antioxidant activities

In the current study, the antioxidant potency of *M. longifolia* extracts has been examined by the following assays, DPPH, TAC, and RP assays.

As displayed in Table 2, the ability to neutralize free radicals (DPPH) showed a significant variation between samples. The cold maceration with 70% ethanol has the highest capacity to scavenge DPPH radicals with (IC_50_ = 0.03 ± 0.00 mg/mL) followed by the same solvent with Soxhlet extraction (IC_50_ = 0.05 ± 0.00 mg/mL). The sonication extraction comes next with an IC_50_ of 0.1 ± 0.0 mg/mL for 70% ethanol. Only water and ethyl acetate in all tested techniques exhibited the lowest DPPH scavenging activity. These results are opposite those obtained by Bourgou et al., who demonstrated the high scavenging ability shown in Soxhlet and sonication extraction with 70% ethanol (IC_50_ = 4.80 and 3.60 μg/mL, respectively) (Bourgou et al. 2016).

Regarding the RP assay, the reducing antioxidant ability was slightly different between the samples tested. The hydroethanolic extract obtained by sonication, and cold maceration extraction shows the highest value of reducing power at 0.07 ± 0.00 and 0.08 ± 0.01 mg/mL, respectively. In contrast, water and ethyl acetate extracts showed the lowest antioxidant capacity. As a result, variations in the antioxidant-reducing power efficiency usually existed among samples. The outcomes were superior to the ones obtained by Tourabi et al. for *M. longifolia* leaves, whose EC_50_ value was 0.26 ± 0.00 mg/mL using cold maceration with 70% ethanol (Tourabi et al. 2023b).

The highest total antioxidant capacities (TAC) were noted in the Soxhlet and cold maceration extractions with 70% ethanol (94.1 ± 1.40 and 93.85 ± 0.01 mg AAE/g DW), proceeded by sonication extraction with the same solvent (88.8 ± 1.8 mg AAE/g DW, aqueous and ethyl acetate extract have slight differences in total antioxidant capacities compared to hydroethanolic extract, these outcomes correspond with the findings provided by Bourgou et al., which revealed that 70% ethanol achieved by Soxhlet extraction showed high total antioxidant capacities (103.0 mg AAE/g DW) (Bourgou et al. 2016).

The achieved data is in harmony with prior results, while reported that the ethanol solvent has a powerful capacity to extract phenolic compounds with higher antioxidant activities (Braga et al. 2008). Therefore 70% ethanol is the solvent that was selected as the most adequate for the extraction of antioxidant components for *M. longifolia*.

To conclude, for obtaining high antioxidant powers of the extracts of *M. longifolia*, the results show that the extraction methods by cold maceration and sonication have strong antiradical activity compared to the Soxhlet extraction with a large superiority of the 70% ethanol solvent. However, the polyphenol content in the Soxhlet extracts was the highest, and the opposite for the extracts achieved by maceration extraction is the richest in flavonoid content.

In addition, a correlation between a boost in antioxidant capability and a high level of TPC and TFC. Although all three extraction methods showed interesting results, maceration, and Soxhlet with 70% ethanol can be selected as the best technique to extract antioxidant compounds from the aerial part of *M. longifolia*.

Maceration is a simple and easier method that has several advantages and a few inconveniences, including, the higher extraction yield, which can be introduced into the maceration technique, when such modifications are necessary, can change the extraction heat, extract the maximum of phytoconstituents (Polyphenols, flavonoids saponins, tannins, and carbohydrates) (Azwanida 2015). As an example, in the case of *Moringa oliefera*, maceration with 70% ethanol revealed the highest content of phenolic components and flavonoids compared to extraction by Soxhlet and percolation with a similar solvent (Vongsak et al. 2013). Furthermore, the cold maceration technique enables the passive diffusion of solvents into plant material, often enhancing antioxidant activity due to the high yield of thermolabile compounds, such as phenolic compounds, achieved without the use of heat (Vongsak et al. 2013).

**Fig. 3:**
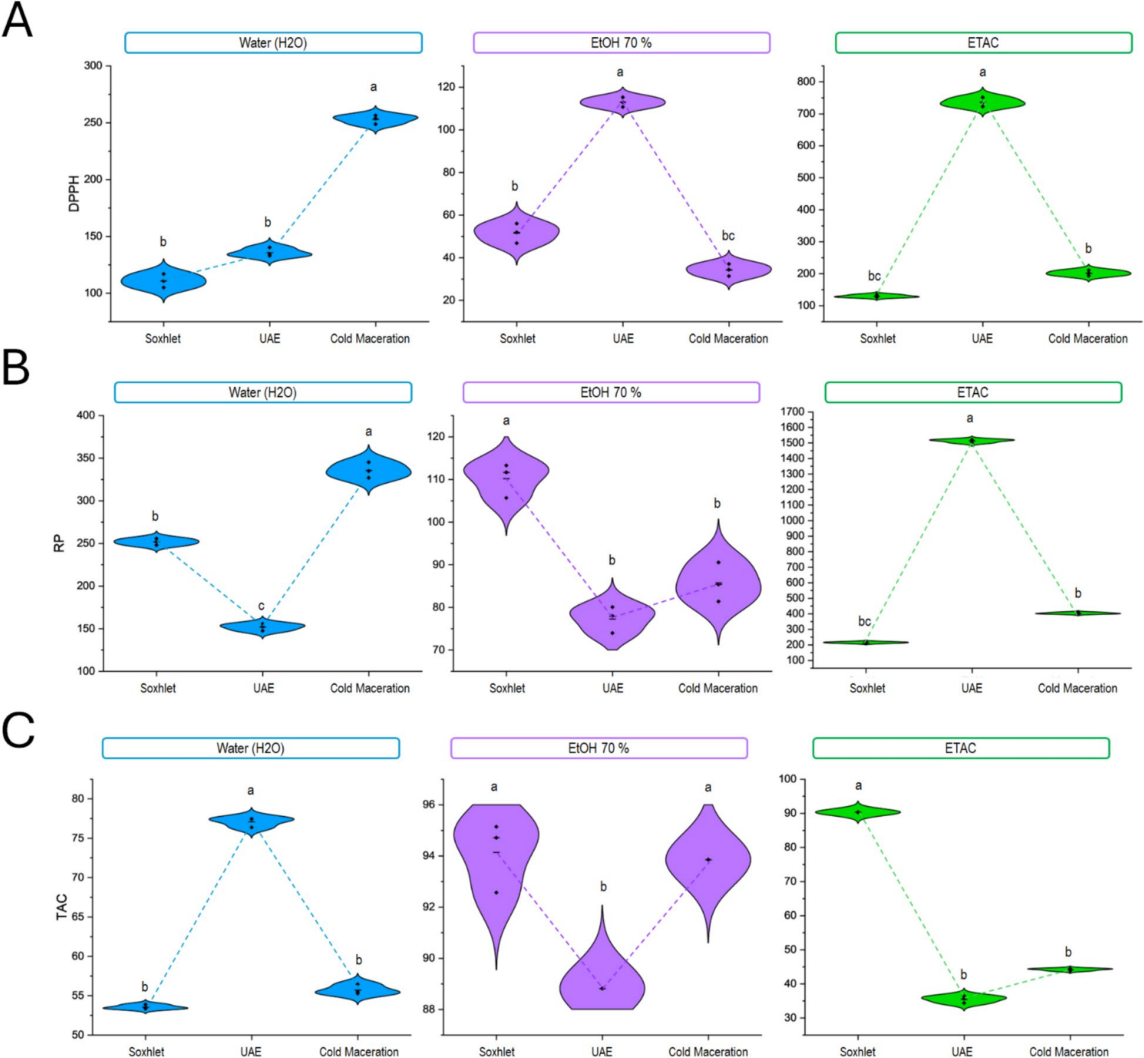
Interaction between the “method × solvent” and the test results; **A**: DPPH; **B**: RP and **C**: TAC on *M. longifolia* extracts

The interaction method × solvent on antioxidant capabilities is illustrated in Fig. 3 respectively for DPPH, RP, and TAC assays. The method × solvent interaction was significant (p < 0.1) for all tests. Additionally, all two-way test interaction "method × solvent" were designated significant for DPPH, RP, and TAC tests (p < 0.001), which differ for each solvent and extraction process. Overall, the results suggest that both the solvent type and the extraction method significantly influence the antioxidant properties of a sample. The specific impact varies depending on the assay used, meaning that optimizing both the solvent and extraction technique is crucial to accurately assess the antioxidant potential of a substance.

### Effect of solvent types and extraction techniques on soluble proteins total soluble carbohydrates and chlorophyll Pigment content.

Protein is an essential macronutrient and a component of the structural composition of several foods (Loveday 2019). The chemical, functional, biological, and nutritional features of proteins in the human diet vary depending on their source, molecular composition, and structures. Our diet's sources of protein are entire foods or prepared foods containing components of fractionated protein derived from either plant or animal sources (Nwachukwu and Aluko 2021).

Moreover, plant proteins have been studied for their potential as functional foods (Hertzler et al. 2020). The food sector has taken a special interest in plant-based proteins because of their greater nutritional value than more proteins generated from animals (Rahman and Lamsal 2021). (Hertzler et al. 2020) This study is the first to estimate the amounts of soluble proteins in our simple.

As regards the soluble protein content of studied extracts, the ultrasonic-assisted extraction (UAE)*,* cold maceration extraction with EtOH 70%, and water solvent have a great amount of soluble protein content 26.5 ± 0.5, 15.22 ± 0.1 mg BSAE/g DW, and 21.91 ± 0.01, 10.81 ± 0.03 mg BSAE/g DW respectively. The Soxhlet extraction indicates the lowest amount of protein content in all solvents tested. The recorded values for soluble protein tenor are greater than those stated by Silva et al. for *Spearmint* whose protein value is 8.9 ± 0.81 μg/g DW obtained by the Soxhlet extraction with water (Silva et al. 2021).

Furthermore, the ultrasonic technique with aqueous ethanol mixtures will increase the extract of more protein than the pure water and ethyl acetate solvent. Likewise, Omura et al. showed that Ultrasonic technology is one of the most efficient techniques to enhance protein extraction, especially when plant proteins are added to food compositions (Omura et al. 2021). However, Soxhlet extraction presented the lowest amount of soluble protein content as a consequence of the high heat which degrades the thermolabile compounds (Seidel 2006).

Carbohydrates are the main source of energy for human nutrition, which promotes body metabolism (Wiesner et al. 2017). The most easily accessed source of energy for the human body is easily available carbohydrates, thus a diet rich in carbohydrates is necessary (Hounsome et al. 2008). According to research, plant-based carbohydrates have many bioactive qualities, such as those that are antibacterial, antidiabetic, and anti-inflammatory, have antioxidant activity, and are anti-cancer (Umavathi et al. 2021).

The obtained findings (Table 3) show that the solubility of carbohydrates differed substantially amongst samples, which the Ultrasonic assisted extraction (UAE), with EtOH 70%, and water showed a high amount of total soluble carbohydrates content with a value of 50.1 ± 0.70, 36.67 ± 0.01 mg/g GE/g DW, and following by Soxhlet extraction with EtOH 70% 26.6 ± 1.4 mg/g GE/g DW, while cold maceration extraction with distilled water have a high amount of carbohydrates content (38.2 ± 0.2 mg/g GE/g DW), Whereas ETAC solvent showed the lowest amount of carbohydrates contained in all extraction process selected. Ralte et al. reported the high carbohydrate content shown in Soxhlet extraction with methanol solvent for *Physalis angulata* L. (35.64 mg/g) (Ralte et al. 2021). Other results showed by Yang et al. indicate the high carbohydrate content showed by ultrasonic extraction with water for *Lycium barbarum* (59.1 ± 2.6%) (Yang et al. 2015).

**Table 3:** Nutritional composition of different *M. longifolia* extracts. The analysis was performed by one-way ANOVA, and the values in the same column with different letters have a significant difference (*p* < 0.05)

Extraction technique	Solvent type	SProtein (mg BSAE/g DW)	Carbohydrate (mg/g EG/g DW)	Ch – a (mg/g DW)	Ch – b (mg/g DW)
Soxhlet	Water	5.6 ± 0.3^b^	7.2 ± 0.2^b^	0.65 ± 0.01^c^	1.47 ± 0.02^c^
	EtOH 70%	7.15 ± 0.15^a^	26.6 ± 1.4^a^	1.4 ± 0.01^b^	3.1 ± 0.02^b^
	ETAC	3.7 ± 0.4^b^	7.75 ± 0.21^b^	4.90 ± 0.00^a^	11.59 ± 0.00^a^
Ultrasonic assisted extraction (UAE)	Water	15.2 ± 0.1^b^	36.67 ± 0.01^b^	1.34 ± 0.00^b^	3.1 ± 0.01^c^
	EtOH 70%	26.5 ± 0.5^a^	50.1 ± 0.70^a^	2.92 ± 0.06^b^	6.54 ± 0.11^b^
	ETAC	0.24 ± 0.03^c^	0.2 ± 0.1^c^	5.56 ± 0.00^a^	12.50 ± 0.0^a^
Cold maceration	Water	10.81 ± 0.03^b^	38.2 ± 0.2^a^	0.72 ± 0.02^b^	1.8 ± 0.04^c^
	EtOH 70%	21.91 ± 0.01^a^	20.8 ± 0.04^b^	4.01 ± 0.0^a^	9.0 ± 0.0^b^
	ETAC	3.8 ± 1.2^c^	0.57 ± 0.39^c^	4.9 ± 0.0^a^	11.6 ± 0.01^a^

Regarding sugars contained in plant extracts, glucose, fructose, sucrose, and fructans are among the water-soluble carbohydrates that constitute the majority of the sugar content of plant extracts (Waite and Boyd 1953). The literature also discusses the solubility of sugars (fructose glucose, sucrose, and lactose,) in a mixture of ethanol, methanol, and water (Peres and Macedo 1997). Besides, the plant contains also insoluble polysaccharides like lignins, cellulose, and some hemicelluloses (Wong et al. 2017). Therefore, the interaction of carbohydrates with other components (protein, polyphenol) may increase the solubility of carbohydrates in aqueous media (Freitas et al. 2003).

The outcomes showed enhancement in the extraction of soluble carbohydrate content using ultrasonic extraction with water and 70% EtOH. Furthermore, Mena-García et al. demonstrate that ultrasonic extraction is the best technique to extract carbohydrates since is simple and effective (increases extraction yield and decreases extraction time), while simultaneously preserving compound structural and molecular characteristics (Mena-García et al. 2019).

Chlorophyll pigments are essential for photosynthesis and responsible for the green color of plants, which also have been part of the human diet for several hundred years (Schoefs 2002). In this section, the content of chlorophyll pigments *a* and *b* were estimated. As shown in Table 3, Ultrasonic assisted extraction (UAE), Cold maceration extraction with 70% EtOH and ETAC, quantified a substantial concentration of chlorophyll pigments *a*, and *b* ranging from 2.92 ± 0.06 to 5.56 ± 0.00 mg/g DW and 6.54 ± 0.11 to 12.50 ± 0.0 mg/g DW respectively for Ultrasonic extraction and varied between 4.01 ± 0.0 to 4.9 ± 0.0 mg/g DW, and 9.0 ± 0.0 to 11.6 ± 0.01 mg/g DW accordingly for Cold maceration extraction. This is lower than previously reported by Zulqarnain et al. which indicates that the high amount of chlorophyll – a value is 14.12 mg/g DW and chlorophyll – b with a concentration of 19.84 mg/g DW obtained by sonication extraction with aqueous ethanol (20/80 v:v) for leaves of *Carica papaya* (Zulqarnain et al. 2021). Whereas Soxhlet extraction with water and 70% EtOH showed the lowest amount of chlorophyll content. Soxhlet extraction, which suggests that the chlorophyll may be degraded as a result of the high heat and the Soxhlet process's length (6 h) (Lefebvre et al. 2020). As a result, the sonication method may be recommended as an effective technique to obtain a higher amount of chlorophyll pigment (Zulqarnain et al. 2021).

### Effect of solvent types and extraction techniques on antimicrobial activity

The antibacterial efficacy of different *M. longifolia* extracts was assessed using a microdilution test, and the results are presented in (Table 4). In the present inquiry, all the analyzed samples revealed wonderful results. Indeed, the EtOH 70%, water, and ethyl acetate extracts of *M. longifolia* achieved by the Soxhlet process demonstrated the strongest antibacterial potential, with a minimum inhibitory concentration varying between 0.19 and 6.25 mg/mL against *E. coli* and *B. subtilis*. Similarly, extracts prepared with ETOH 70% and ETAC achieved by cold maceration process exhibited a high inhibition of growth of *E. coli* with a MIC value of 0.39 mg/mL. The sensitivity of microorganisms to various extracts tested for their antimicrobial properties is evaluated, and it is discovered that the *E. coli* strain exhibits the highest sensitivity, immediately followed by *B. subtilis*, where *P. aeruginosa* exhibits an intermediate sensitivity, and *S. aureus* exhibits minimal sensitivity. Furthermore, extracts prepared with 70% ethanol had higher antibacterial activity than extracts obtained with ethyl acetate and water.

**Table 4:** Antibacterial potential of different *M. longifolia* extracts, using MIC, MBC, and MBC/MIC tests

		*Mentha longifolia*											
		Gam-negative bacteria						Gram-positive Bacteria					
		*P. aeruginosa*			*E. coli*			*S. aureus*			*B. subtilis*		
		MIC	MBC	MBC/MIC	MIC	MBC	MBC/MIC	MBC	MBC/MIC	MIC	MBC/MIC	MIC	MBC
Soxhlet	Water	6.25	6.25	1	0.19	ND	–	12.50	12.50	1	0.78	ND	–
	ETOH 70%	6.25	6.25	1	0.19	ND	–	6.25	6.25	1	3.12	ND	–
	ETAC	6.25	6.25	1	0.19	ND	–	3.12	3.12	1	6.25	ND	–
Ultrasonic assisted extraction (UAE))	Water	50.00	ND	–	0.78	ND	–	ND	ND	–	–	ND	–
	ETOH 70%	6.25	ND	–	0.78	ND	–	6.25	50.00	8	1.56	ND	–
	ETAC	6.25	6.25	1	3.12	ND	–	6.25	6.25	1	1.56	ND	–
Cold maceration	Water	50.00	ND	–	ND	ND	–	ND	ND	–	–	ND	–
	ETOH 70%	6.25	6.25	1	0.39	ND	–	6.25	6.25	1	1.56	1.56	–
	ETAC	6.25	12.50	2	0.39	ND	–	6.25	50.00	8	3.12	ND	–

Minimum bactericide concentration (MBC), minimum inhibitory concentration (MIC), and not determined (ND)

The variations in the composition of extract produced by the use of different solvent polarities may be the reason for this variation in antibacterial efficacy.

The examined extracts demonstrated that the MBC/MI ratio was less than 4, indicating an effective bactericidal effect on *P. aeruginosa* and *S. aureus* strains. Based on the findings extracts with a CMB/MIC ratio of 4 or less are more likely to have bactericidal activity, while those with a ratio higher than 4 are more probably to have bacteriostatic action (Konaté et al. 2012). Within the context of a study conducted by Babakhani and colleagues (Babakhani et al. 2016), the ethanolic extract of *Mentha pulegium* obtained by Soxhlet extraction demonstrated a significant antibacterial activity compared to that observed for our extracts, with MIC values of 62.50 µg/mL for *S. aureus*, 3.90 µg/mL for *B. cereus*, 15.62 µg/mL for *E. coli*, and 31.25 µg/mL for *P. aeruginosa.* Additionally, our findings align with those of Katarzyna Rafińska, who reported a MIC value of 1 mg/mL for the supercritical fluid extraction extract from freeze-dried *Lepidium sativum* sprouts against *E. coli* (Rafińska et al. 2019).

Regarding antifungal activity, the majority of tested extracts of *M. longifolia* demonstrated effectiveness against the two fungal strains studied. The corresponding results are presented in (Table 5). Indeed, the hydroethanolic extract obtained by the Soxhlet method as well as the extraction by cold maceration demonstrated a varied and extensive antifungal capacity with an MIC varying between 6.25 and 12.50 mg/mL against *C. albicans* and *A. niger*, as well as characterized by MFC/MIC ratios less than or equal to (≤ 4). Similarly, ETAC extract achieved by sonication technique exhibited a bactericidal effect against both fungi with an MFC/MIC ratio less than or equal to (≤ 4).

**Table 5:** Antifungal effect of different *M. longifolia* extracts using MIC, MFC, and MFC/MIC tests

Mentha longifolia							
		*C. albicans*			*A. niger*		
		MIC	MFC	MFC/MIC	MIC	MFC	MFC/MIC
Soxhlet	Water	50.00	ND	ND	ND	ND	–
	ETOH 70%	6.25	6.25	1	6.25	6.25	1
	ETAC	6.25	12.50	2	6.25	12.5	2
Ultrasonic assisted extraction (UAE))	Water	ND	ND	–	ND	ND	–
	ETOH 70%	12.50	12.50	1	6.25	12.50	2
	ETAC	6.25	12.50	2	6.25	ND	–
Cold maceration	Water	ND	ND	–	50.00	50.00	1
	ETOH 70%	6.25	6.25	1	6.25	12.50	2
	ETAC	6.25	50.00	8	6.250	ND	–

*ND* Not determined

The ETOH 70% and ETAC extracts obtained by Soxhlet and cold maceration procedure showed the strongest antimicrobial activity. The significant antimicrobial effects observed in our study are likely due to the ability of 70% ethanol (ETOH) and ethyl acetate (ETAC) solvents to extract antimicrobial compounds, dissolving both hydrophilic and lipophilic components. Indeed, this activity might be explained via its rich phytochemical composition, especially rosmarinic acid, gallic acid, ferulic acid, kaempferol, quercetin, and rutin. Likewise, various reports stated the antimicrobial efficiency of phenolic acids (rosmarinic acid, gallic acid, ferulic acid,) as well as flavonoids (kaempferol, quercetin, and rutin). These bioactive molecules have been stated to have great antibacterial potential through several biological pathways, such as the creation of reactive quinones, hyper-permeabilization of membranes, hyper-acidification, and barrier destabilization. Thus, Johnston et al., equally, reported that the phenolic acids especially hydroxycinnamic acids (caffeic and ferulic acids) diffuse passively inside the bacterial cell by enhancing the permeability of the cytoplasmic membrane which induced leakage in the composites of the bacterial cell such as nucleic acid, as well as inorganic ions (P/K) (Johnston et al. 2003). The strongest antibacterial effect versus *S. aureus* and *E. coli* has been recorded for quercetin and kaempferol, with MIC values of 125 and 62 µg/mL, accordingly. The attained inhibitory activity could be made via both mechanisms, first blocking cell wall synthesis, and second inhibiting the cell membrane production (Resende et al. 2016). In the same way, gallic acid has been revealed to provoke bacterial cell death in the same context by changing the bacterial cytoplasm and possibly acidifying it through increased K + discharge and degradation of proteins. These changes can cause the expulsion of components from inside cells, alter the permeability of the cytoplasmic barrier, and damage walls (Campos et al. 2003).

### Effect of extraction method and solvent types on extraction yield, chemical properties and antioxidant activity

**Fig. 4:**
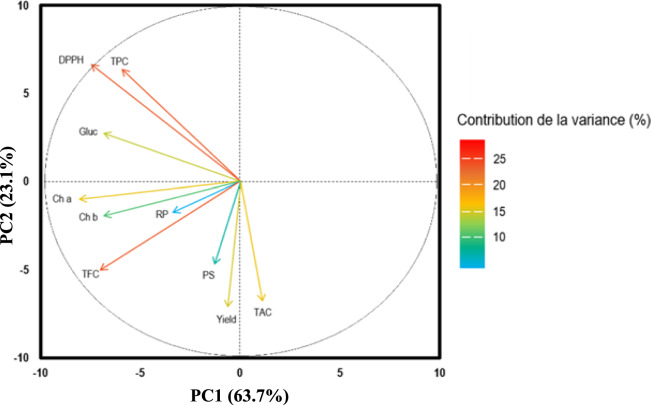
Main Component Analysis (PCA) shows the correlations between antioxidant activities, the content of TPC, TFC, PS, Carbohydrates, and Ch – a and Ch – b of different *M. longifolia* extracts

To illustrate how the extraction technique and solvent type altered the *M. longifolia* extracts' yield, chemical makeup, and antioxidant activity, a principal component analysis (PCA) was executed (Fig. 4). The two principal components of Fig. 4, PC1 and PC2, represented the majority of the variance, representing 63.7% and 23.1% respectively. Variables that contribute little to the distinction of the extracts will be located near the origin of the chart, while those that have a more significant contribution will be situated further away from the center of the plot. The variables that have the highest contribution to extract differentiation are: antioxidant activity (DPPH: 25.86%), TFC (23.55%), and TPC (22.04%), the variables with the middle contribution are those correlated with TAC (17.66%), photosynthetic pigments (chlorophyll– a and chlorophyll – b 16.80%, and 11.79% respectively), the extraction the yield (15.57%), and carbohydrate content 13.93%. The variables with lower contribution were, protein content (8.96%), and reducing power (RP: 1.38%) as illustrated in Fig. 4. The results indicate that the samples produced had relatively consistent reducing power and protein content. However, notable variations were observed in phenolic content, antioxidant activity, carbohydrate levels, photosynthetic pigment concentrations, and extraction yield.

Figure 4 reveals correlations between variables, with positively associated variables appearing close to each other and negatively correlated variables appearing diagonally opposite. The PCA indicates that TPC and TFC are positively correlated with antioxidant activity, likely due to phenolic compounds' redox properties, which facilitate free radical adsorption and neutralization. Studies on various plant materials have shown a strong correlation between phenolic compounds and antioxidant activity (Silva et al. 2021).

## Conclusion

The current experimentation offers new results indicating that the extraction technique and the solvent polarities altered the majority of the chemical properties and antioxidant profile as well as the antimicrobial potential of the samples. The results also suggest interactions involving the type of solvent and the technique of extraction, influencing various chemical compositions and antioxidant properties of the extracts. Additionally, ETOH 70% was especially efficient in the extraction of polyphenolic compounds from *M. longifolia* leaf extract. On the other hand, cold maceration combined with ETOH 70% extracted mainly flavonoids. The cold maceration combined with 70% ethanol has the highest capacity to scavenge DPPH radicals with (IC_50_ = 0.03 ± 0.00 mg/mL), the UAE has a great reducing power with EC_50_ = 0.07 ± 0.00 mg/mL, while the highest total antioxidant capacities (TAC) were noted in the Soxhlet and maceration extraction with 70% ethanol (94.1 ± 1.40 and 93.85 ± 0.01 g AAE/g DW) respectively. The UAE with ETOH showed important nutritional properties, like soluble carbohydrate, and soluble protein, while aqueous extract obtained by use of UAE extraction maximized pigment content. The best antibacterial activity was observed for the extract of ETOH 70%, which was obtained by the use of Soxhlet and maceration processes with a MIC value of 0.19 mg/ mL and 0.39 mg/mL respectively against *E. coli*. HPLC–DAD analysis displays that Soxhlet with ETOH 70% was the most selective extraction technique for recovering molecules with high bioactivities. Based on the current study, *Mentha longifolia* exhibits significant antimicrobial activity, suggesting its potential use in treating gastrointestinal and fungal infections. Additionally, its strong antioxidant properties, illustrated through high radical scavenging activity, imply that it could be beneficial in managing metabolic disorders such as diabetes and cardiovascular diseases by reducing oxidative stress and improving overall metabolic function. Overall, the rich phenolic composition, nutritional content, and performant properties of *M. longifolia* extracts indicate that it could potentially be used as a multifaceted biofunctional agent in the food, pharmaceutical, or nutraceutical sectors.

This study could be reinforced through more comprehensive research, notably incorporating sustainable and environmentally friendly approaches, such as supercritical fluid extraction, and microwave-assisted extraction, integration of multi-objective optimization such as the polarity and solvent ratio, temperature, exploration of novel solvents (e.g.: deep eutectic solvents and ionic liquids), as well as future studies should address the scalability of optimized processes to ensure feasibility for industrial applications.

## Data Availability

All data generated or analyzed during this study are included in this published article.
