# Correction: Optimization of extraction process and solvent polarities to enhance the recovery of phytochemical compounds, nutritional content, and biofunctional properties of *Mentha longifolia* L. extracts

**DOI:** 10.1186/s40643-025-00910-8

**Published:** 2025-07-18

**Authors:** Meryem Tourabi, Khaoula Faiz, Rachid Ezzouggari, Bouchra Louasté, Mohammed Merzouki, Musaab Dauelbait, Mohammed Bourhia, Khalid S. Almaary, Farhan Siddique, Badiaa Lyoussi, Elhoussine Derwich

**Affiliations:** 1 Laboratory of Biotechnology, Conservation and Valorization of Bioresources, Faculty of Sciences, Sidi Mohamed Ben Abdellah University, Fez, Morocco; 2 Laboratory of Biotechnology, Environment, Agri-Food, and Health, Faculty of Sciences Dhar El Mahraz, Sidi Mohamed Ben Abdellah University, Fez, Morocco; 3 Phytopathology Unit, Department of Plant Protection, Ecole Nationale d’Agriculture de Meknès, Km10, Rte Haj Kaddour, BP S/40, 50001 Meknès, Morocco; 4 Unity of GC/MS and GC-FID, City of Innovation, Sidi Mohamed Ben Abdellah University, Fez, Morocco; 5 University of Bahr El Ghazal, Freedowm Stree, Wau, 91113 South Sudan; 6 Laboratory of Biotechnology and Natural Resources Valorization, Faculty of Sciences, Ibn Zohr University, 80060 Agadir, Morocco; 7 Department of Botany and Microbiology, College of Science, King Saud University, P. O. BOX 2455, 11451 Riyadh, Saudi Arabia; 8 School of Pharmaceutical Science and Technology, Tianjin University, Tianjin, People’s Republic of China

**Correction to: Bioresources and Bioprocessing (2025) 12:24** 10.1186/s40643-025-00859-8

In this article the wrong figure appeared as Fig. 4; the figure should have appeared as shown below.

Incorrect Fig. 4
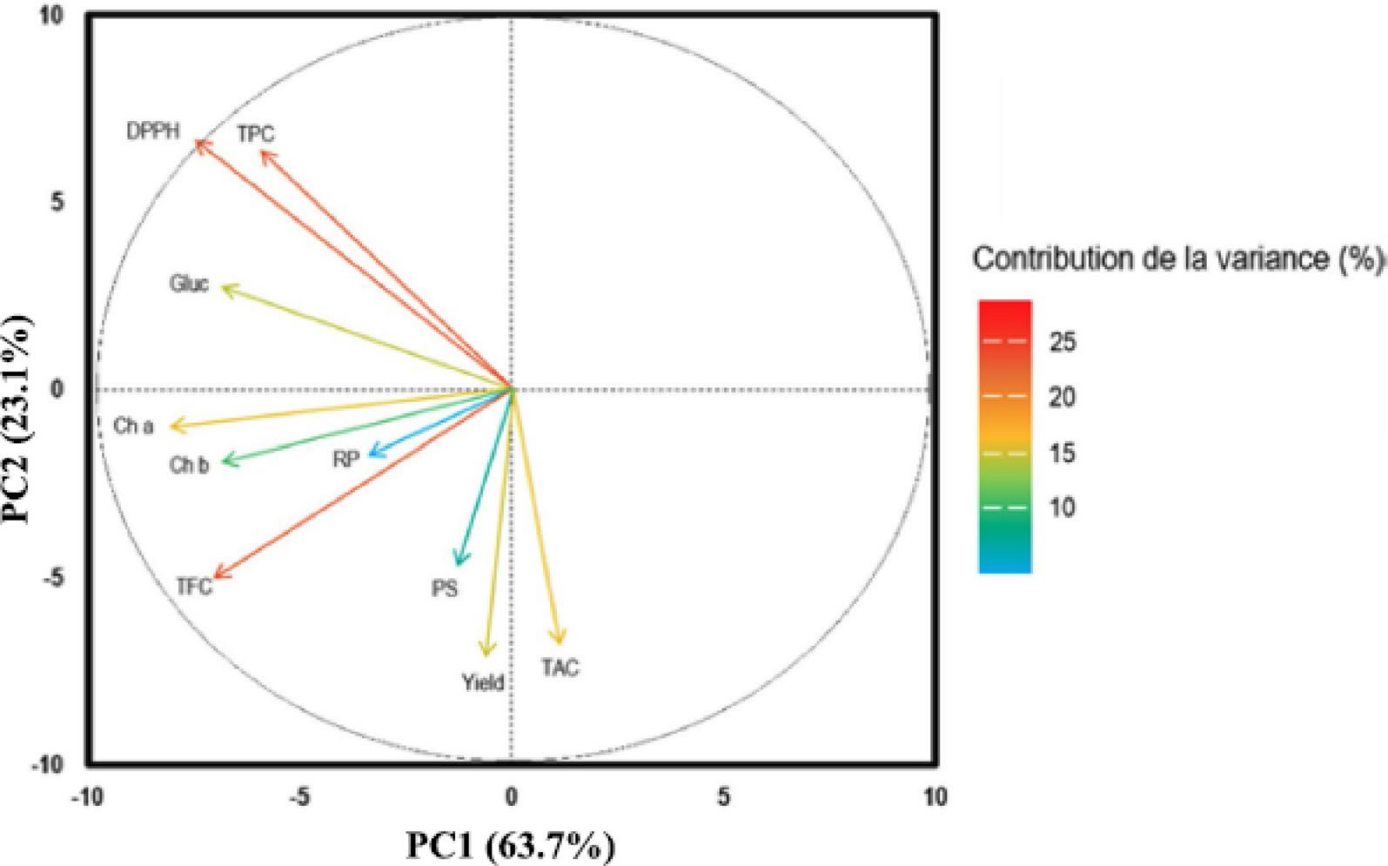


Correct Fig. 4

**Fig. 4:**
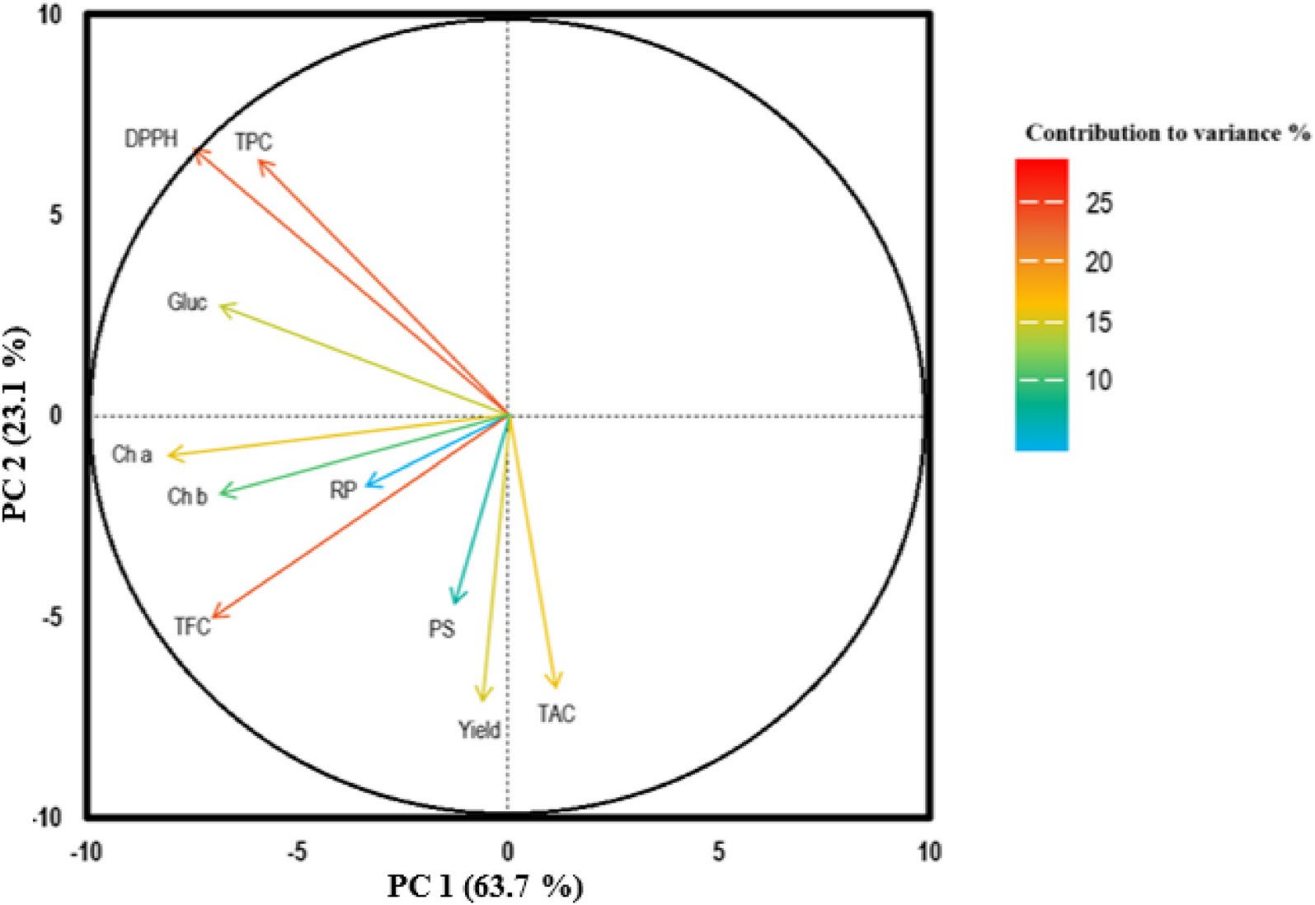
Main Component Analysis (PCA) shows the correlations between antioxidant activities, the content of TPC, TFC, PS, carbohydrates, and Ch – a and Ch – b of different *M. longifolia* extracts

